# Supplementary material for: Hypoxia enhances antibody‐dependent dengue virus infection
Source: EMBO J. 2017 Mar 20;36(10):1348–63. doi: 10.15252/embj.201695642 (PMC5430213; doi:10.15252/embj.201695642)
Supplement: Supplementary file 1 — Appendix [file EMBJ-36-1348-s001.pdf]

Appendix

Table of Contents

Legends……………………. 2

Appendix Figure S1: FcγRIIA antibody for flow cytometry is specific to FcγRIIA... 4

Appendix Figure S2: Increase in DENV RNA observed at 0.5% O_2 5

Appendix Table S1: TBP mRNA levels at various time points post O_2 adaptation . 6

Appendix Table S2: HIF1α ChIP-Seq unable to enrich DNA under normoxic conditions 7

Appendix Table S3: List of PE lipids in Fig 5a. 8

Appendix Table S4: List of ether and plasmalogen PE lipids in Fig 5a. 9

Legends

Appendix Figure S1: FcγRIIA antibody for flow cytometry is specific to FcγRIIA.

Mean fluorescence intensity (MFI) of FcγRIIA on THP-1 monocytes was assessed by flow cytometry. FcγRIIA blocking polyclonal antibodies reduced the MFI observed compared to no block conditions. Isotope antibodies were used as a control and showed minimal fluorescence.

Appendix Figure S2: Increase in DENV RNA observed at 0.5% O_2

a,b. Culturing THP1 cells at 3% (a) and 0.5% (b) results in the increase in DENV RNA 18hpi under ADE conditions as compared to 20% O_2. **c.** mRNA levels of VEGF increases 24h post oxygen adaptation at 0.5% O_2 as compared to 20% O_2.

Appendix Table S1: TBP mRNA levels at various time points post O_2 adaptation

Levels of TBP mRNA at different time points was assessed by full genome microarray and normalized by quantile normalization. Ratio was calculated by (TBP mRNA in hypoxic conditions / TBP mRNA in normoxic conditions). TBP mRNA levels are constant under both hypoxic and normoxic conditions across time points.

Appendix Table S2: HIF1α ChIP-Seq unable to enrich DNA under normoxic conditions

Concentrations of DNA enriched after HIF1α and control Rabbit IgG or Histone H3 ChIP. No DNA was detected with Qubit DNA quantification assays using

control Rabbit IgG or HIF1α antibody under normoxic conditions. High concentrations of DNA was enriched with Histone H3 antibodies.

Appendix Table S3: List of PE lipids in Fig 5a.

Fold change was calculated by (mean hypoxia / mean normoxia). Genes upregulated in hypoxia is expressed as a positive value and genes upregulated in normoxia is expressed as a negative value.

Appendix Table S4: List of ether and plasmalogen PE lipids in Fig 5a.

Fold change was calculated by (mean hypoxia / mean normoxia). Genes upregulated in hypoxia is expressed as a positive value and genes upregulated in normoxia is expressed as a negative value.

Appendix Figure S1: FcγRIIA antibody for flow cytometry is specific to FcγRIIA.

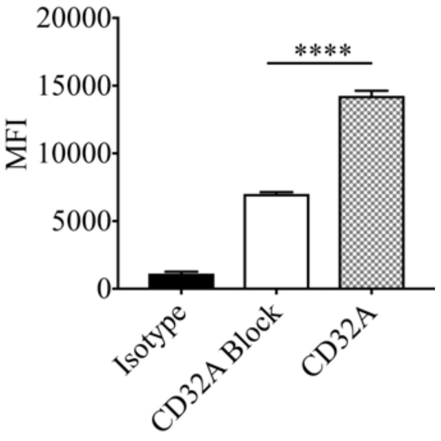

Appendix Figure S2: Increase in DENV RNA observed at 0.5% O_2

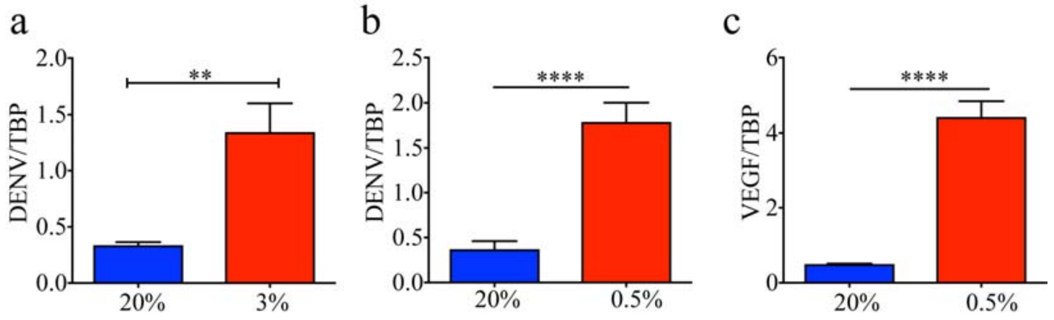

Appendix Table S1: TBP mRNA levels at various time points post O_2 adaptation

Hours Post Oxygen Adaptation	Ratio (3% / 20%)
24	0.94
26	0.96
30	0.99
48	1.00

Appendix Table S2: HIF1α ChIP-Seq unable to enrich DNA under normoxic conditions

Conditions	Concentration (ng/μl)
2% Input	4.98
Histone H3 (D2B12)	16
Rabbit IgG	-
HIF1α 20% O_2	-

Appendix Table S3: List of PE lipids in Fig 5a.

	Lipid Species	Fold Change
**PE**	PE 32:2	-1.7459
	PE 32:1	-1.3925
	PE 32:0	-1.5746
	PE 34:3	-1.3399
	PE 34:2	-1.4315
	PE 34:1	-1.5029
	PE 34:0	-1.5943
	PE 36:5	-1.4185
	PE 36:4	-1.2842
	PE 36:3	-1.2904
	PE 36:2	-1.4817
	PE 36:1	-1.6803
	PE 38:6	-1.2714
	PE 38:5	-1.3118
	PE 38:4	-1.2652
	PE 38:3	-1.5109
	PE 38:2	-1.6514
	PE 40:7	-1.3983
	PE 40:6	-1.4862
	PE 40:5	-1.3479
	PE 40:4	-1.5681
	PE 40:3	-1.4451
	PE 40:2	-1.8935
	PE 40:1	-1.9311
	PE 42:2	-2.0270
	PE 42:1	-1.8937

Appendix Table S4: List of ether and plasmalogen PE lipids in Fig 5a.

	Lipid Species	Fold Change
Ether PE	PE(O-32:1)/oddPE 31:1	2.8134
	PE(O-32:0)/oddPE 31:0	1.1186
	PE(O-34:2)/oddPE 33:2	4.2384
	PE(O-34:1)/oddPE 33:1	2.3325
	PE(O-36:5)/oddPE 35:5	3.2295
	PE(O-36:4)/oddPE 35:4	2.8498
	PE(O-36:3)/oddPE 35:3	3.2612
	PE(O-36:2)/oddPE 35:2	3.0171
	PE(O-38:6)/oddPE 37:6	1.1546
	PE(O-38:5)/oddPE 37:5	2.3505
	PE(O-38:4)/oddPE 37:4	2.4332
	PE(O-40:7)/oddPE 39:7	1.4622
	PE(O-40:6)/oddPE 39:6	1.1898
	PE(O-40:5)/oddPE 39:5	2.6066
Plasmalogen PE	PE(P-16:0/16:0)	-1.3746
	PE(P-16:0/18:2)	-1.3786
	PE(P-16:0/18:1)	-1.5715
	PE(P-18:0/16:0)	-1.9608
	PE(P-16:0/20:5)	-1.4114
	PE(P-16:0/20:4)	-1.3522
	PE(P-18:1/18:2)	-1.4764
	PE(P-16:0/20:3)	-1.4885
	PE(P-18:0/18:2)	-1.6152
	PE(P-18:0/18:1)	-2.1307
	PE(P-16:0/22:6)	-1.3955
	PE(P-18:1/20:4)	-1.8174
	PE(P-16:0/22:5)	-1.6159
	PE(P- 18:0/20:4)	-1.8956
	PE(P-18:0/20:3)	-2.0291
	PE(P-18:0/22:6)	-1.8189
	PE(P-18:0/22:5)	-2.2268
	PE(P-20:0/20:4)	-1.5000
	PE(P-18:0/22:4)	-2.0019
