# Supplementary material for: Hypoxia enhances antibody‐dependent dengue virus infection
Source: EMBO J. 2017 Mar 20;36(10):1348–63. doi: 10.15252/embj.201695642 (PMC5430213; doi:10.15252/embj.201695642)
Supplement: Supplementary file 2 — Expanded View Figures PDF [file EMBJ-36-1348-s002.pdf]

Expanded View Figures

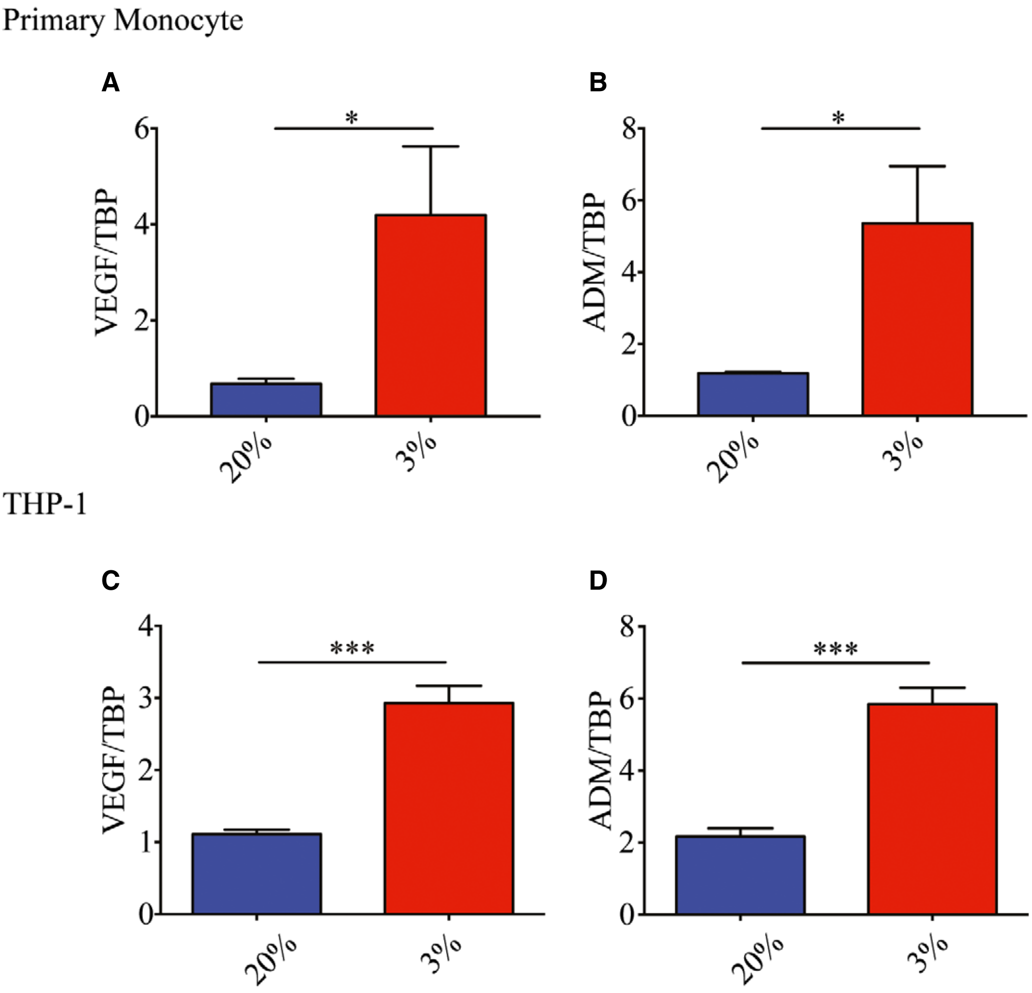

Figure EV1. Incubation at 3% oxygen upregulates expression of known hypoxia-inducible genes in human primary monocytes and THP-1 cells.

A, B Vascular endothelial growth factor (VEGF) (A) and adrenomedullin (ADM) mRNA (B) expression in primary monocytes 24 h post-culture in 3% O_2 (n = 4 biological replicates for each condition).

C, D Vascular endothelial growth factor (C) and ADM mRNA (D) expression in THP-1 cells 24 h post-culture in 3% O_2 (n = 4 biological replicates for each condition).

Data information: Data are presented as mean ± SD. *$P < 0.05$, ***$P < 0.001$ (unpaired t-test).

© 2017 The Authors

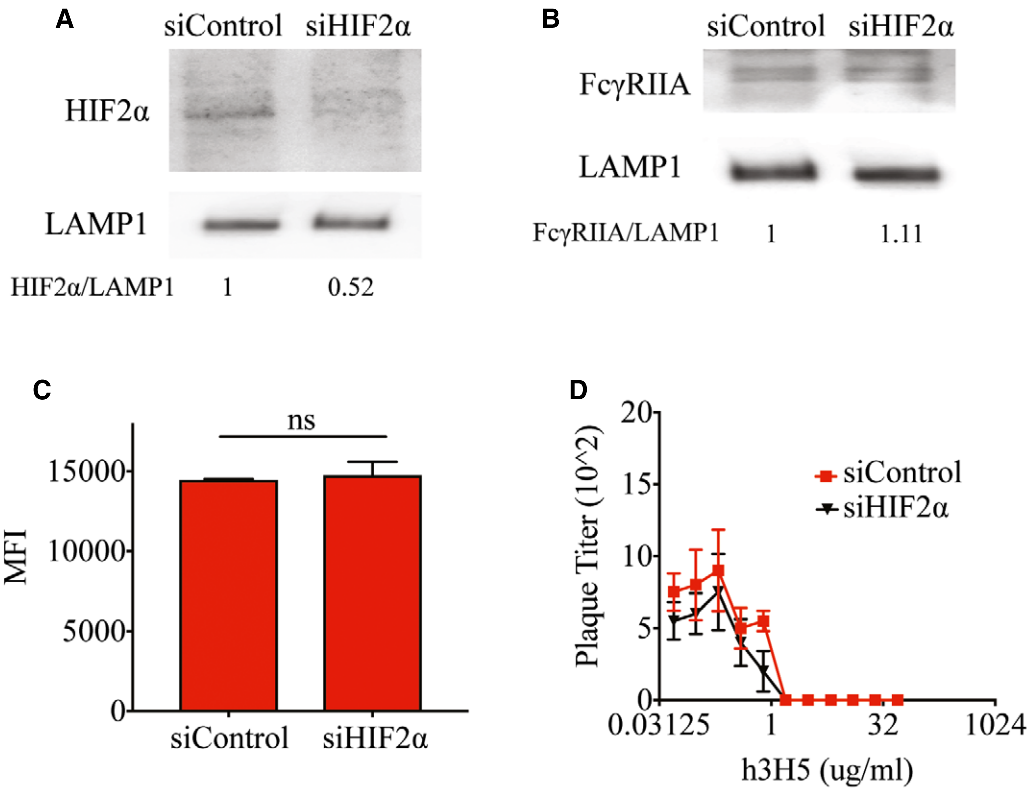

Figure EV2. Hypoxia-induced increase in FcγRIIA expression and DENV infection is independent of HIF2α.

A Knockdown efficiency of HIF2α in control and HIF2α-silenced THP-1 cells cultured at 3% O_2 assessed by Western blot. LAMP1 served as a loading control. Numbers under the Western blot indicate levels of HIF2α normalized to LAMP1.

B Protein levels of FcγRIIA in control and HIF2α-silenced THP-1 cells cultured at 3% O_2 assessed by Western blot showed no difference in FcγRIIA expression. LAMP1 served as a loading control. Numbers under the Western blot indicate levels of FcγRIIA normalized to LAMP1.

C Control and HIF2α-silenced THP-1 cells cultured at 3% O_2 were infected with DENV-2 opsonized with enhancing concentrations of h3H5 (0.391 μg/ml). Internalization was assessed at 2 hpi by flow cytometry revealing no changes in internalization in HIF2α-silenced THP-1 cells as compared to control cells (n = 4 biological replicates for each condition).

D Control and HIF2α-silenced THP-1 cells cultured at 3% O_2 infected with DENV-2 opsonized with varying concentrations of h3H5 antibody showed no significant difference in both neutralization and enhancement profiles (n = 4 biological replicates for each condition).

Data information: In (C, D) data are presented as mean ± SD (unpaired t-test).

Source data are available online for this figure.

 © 2017 The Authors

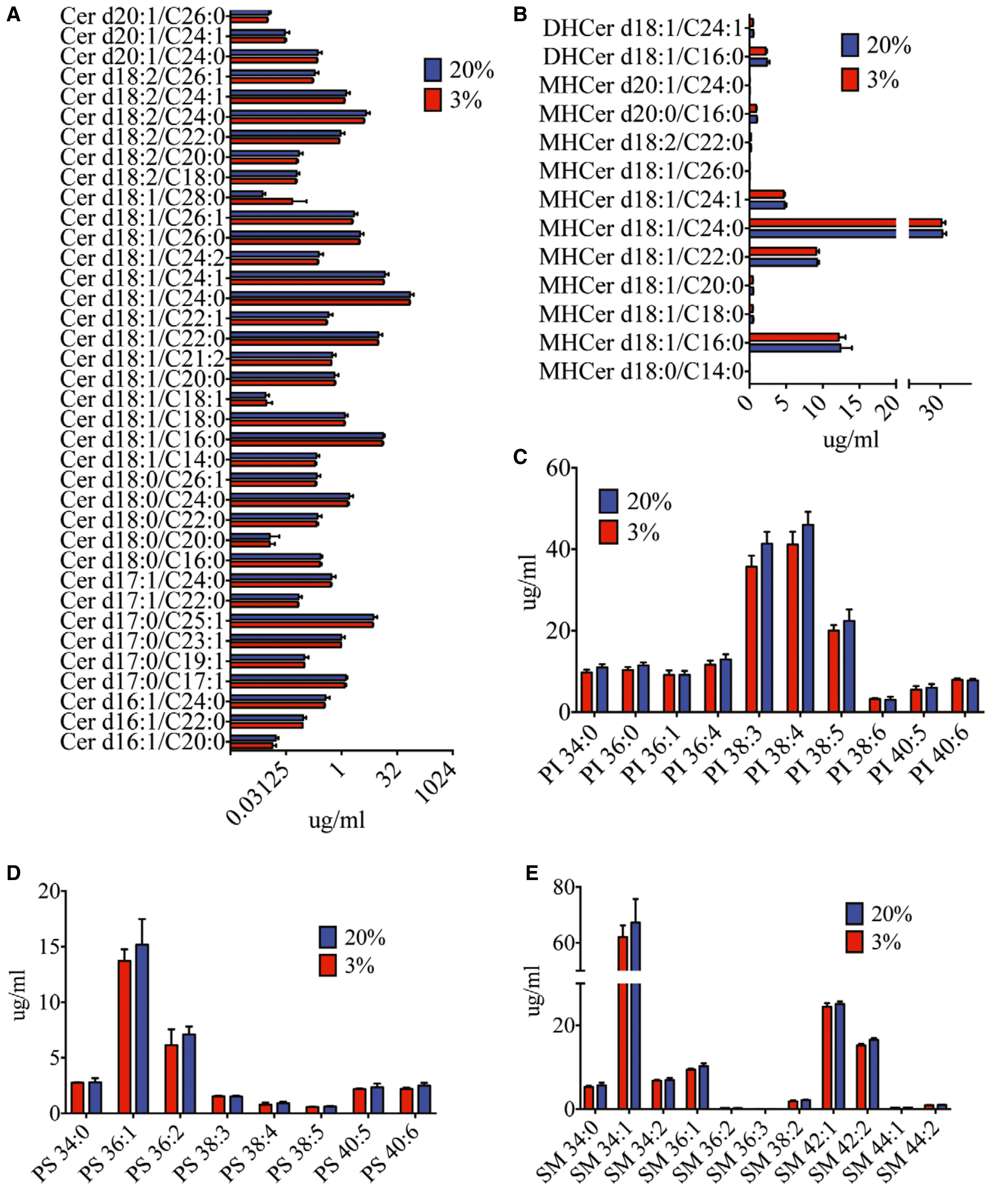

Figure EV3. Lipid profile of THP-1 cells after 24 h of oxygen adaptation.

A–E No significant differences were observed in lipid profiles of ceramide (Cer) (A), dihexosylceramide (DHCer), monohexosylceramide (MHCer) (B), phosphatidylinositol (PI) (C), phosphatidylserine (PS) (D), and sphingomyelin (SM) (E) in hypoxic and normoxic THP-1 cells. Blue and red bars indicate normoxia and hypoxia, respectively, in all panels (n = 3 biological replicates for each condition). Data are presented as mean ± SD (unpaired t-test).

© 2017 The Authors

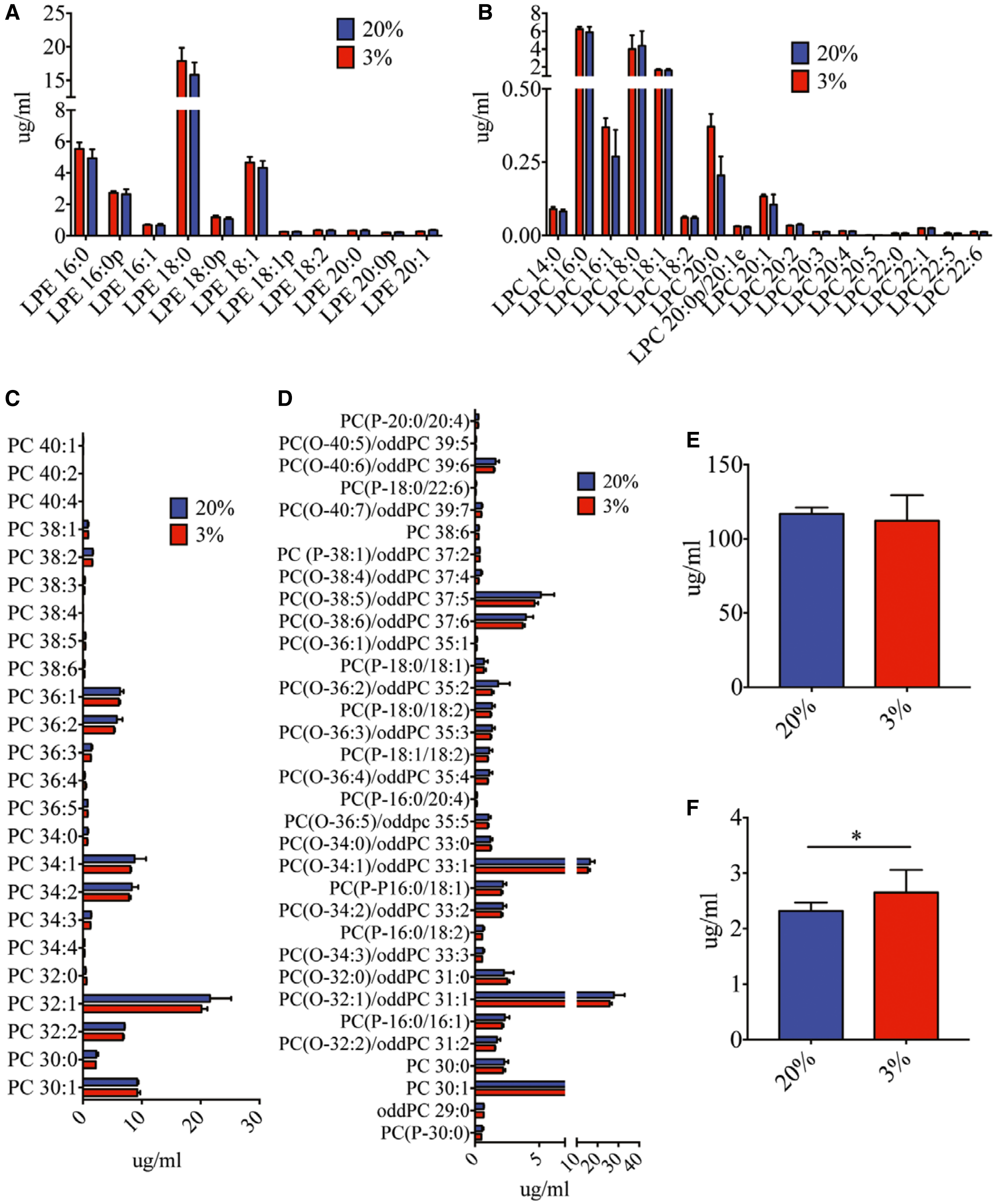

Figure EV4. Lipid profiles of THP-1 cells post-oxygen adaptation.

A–F No significant differences were observed in lipid profiles of lyso-phosphatidylethanolamine (LPE) (A), lyso-phosphatidylcholine (LPC) (B), phosphatidylcholine (PC) (C), plasmalogen (PC(-P)) and ether phosphatidylcholine (PC(-O)) (D), and cholesterol (E) 24 h post-hypoxic and normoxic adaptation. Cholesterol esters (F) were slightly upregulated under hypoxic conditions. Blue and red bars indicate normoxia and hypoxia, respectively, in all panels (n = 3 biological replicates for each condition). Data are presented as mean ± SD. *$P < 0.05$ (unpaired t-test).

© 2017 The Authors

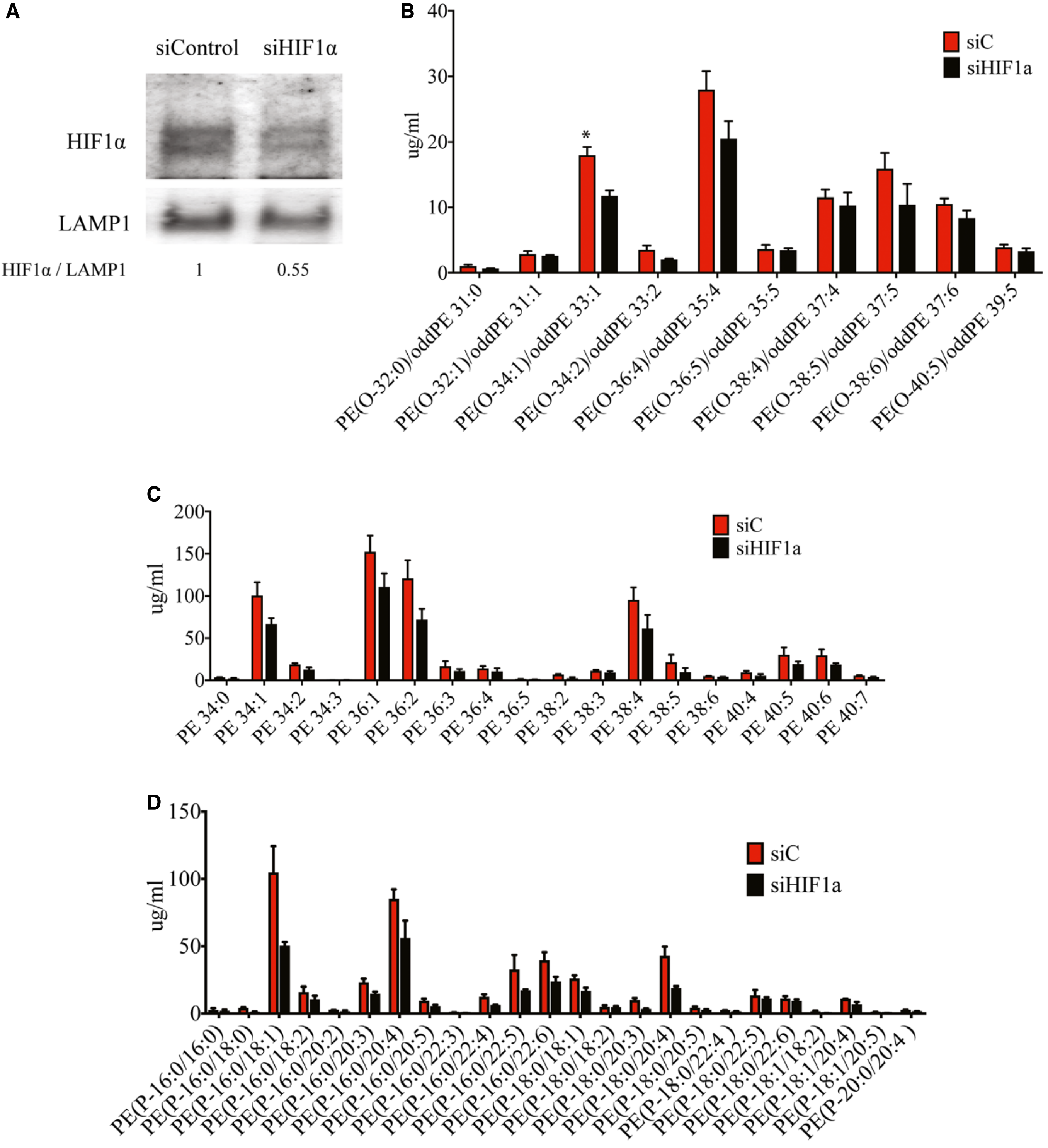

Figure EV5. Hypoxia-induced increase in ether PE lipids is independent of HIF1α.

A Knockdown efficiency of HIF1α in control and HIF1α-silenced THP-1 cells cultured at 3% O_2 assessed by Western blot. LAMP1 served as a loading control. Numbers under Western blot indicate levels of HIF1α normalized to LAMP1.

B–D No significant differences in ether-linked PE concentrations (B), PE concentrations (C), and plasmalogen PE concentrations (D) in control and HIF1α-silenced THP-1 cells cultured at 3% O_2 for 24 h (n = 3 biological replicates for each condition).

Data information: Data are presented as mean ± SD. *$P < 0.05$ (unpaired t-test).

Source data are available online for this figure.

© 2017 The Authors
