# Supplementary material for: Hypoxia enhances antibody‐dependent dengue virus infection
Source: EMBO J. 2017 Mar 20;36(10):1348–63. doi: 10.15252/embj.201695642 (PMC5430213; doi:10.15252/embj.201695642)
Supplement: Supplementary file 3 — Movie EV1 [file EMBJ-36-1348-s003.zip › Movie_EV1_Legend.docx]

**Legend**

**Movie EV1: FcγRIIA directly mediates internalization of DENV immune complexes.**

Hypoxic THP-1 cells were infected with DENV immune complexes and imaged 1-hour post infection. Imaris imaging software was used to reconstruct the plasma membrane (blue) and identify internalized FcγRIIA (red) interacting with DENV (green).
