# Supplementary figures and images for: Hypoxia enhances antibody‐dependent dengue virus infection
Source: EMBO J. 2017 Mar 20;36(10):1348–63. doi: 10.15252/embj.201695642 (PMC5430213; doi:10.15252/embj.201695642)

Figure EV2: Source Data

A

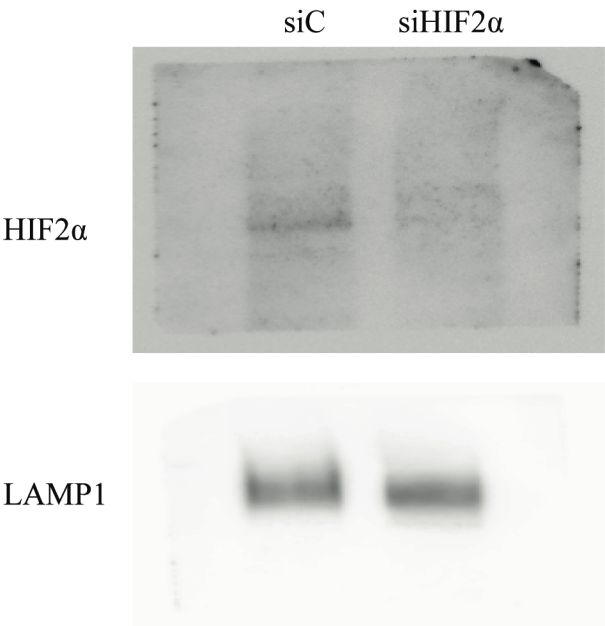

B

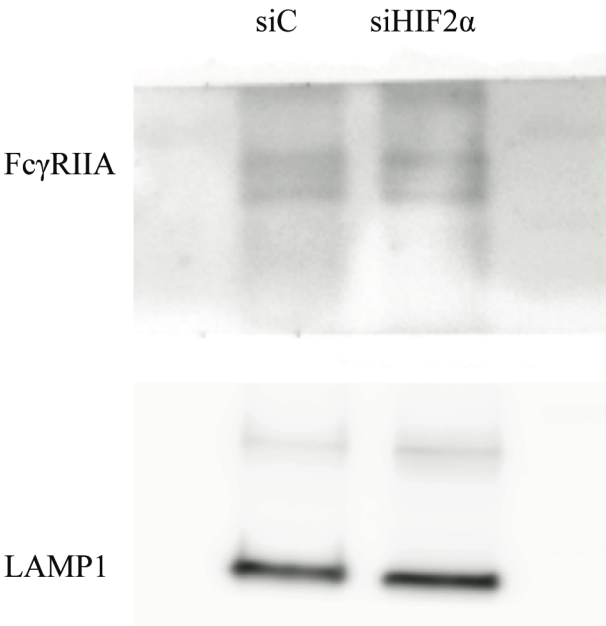

Supplement: Supplementary file 4 — Source Data for Expanded View [file EMBJ-36-1348-s008.zip › Source_Data_EV_Figures/Source_Data_FigureEV2.pdf]

Figure EV5: Source Data

A

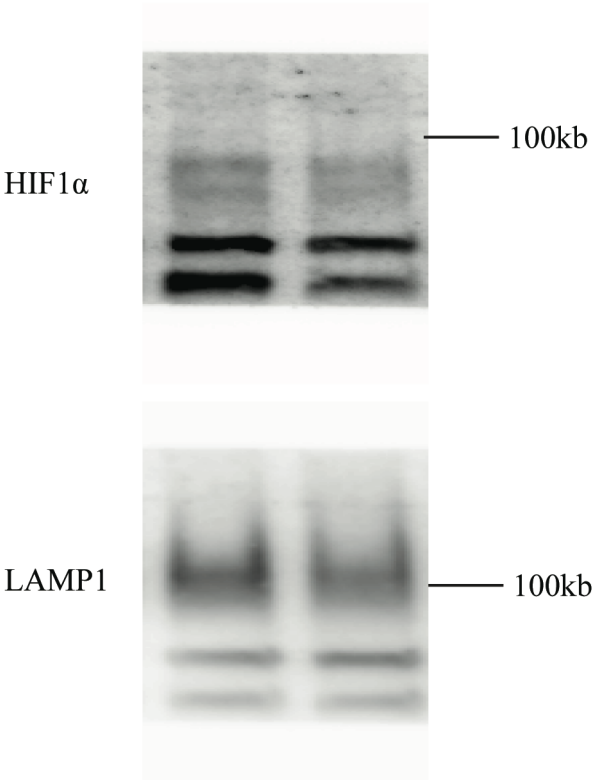

Supplement: Supplementary file 4 — Source Data for Expanded View [file EMBJ-36-1348-s008.zip › Source_Data_EV_Figures/Source_Data_FigureEV5.pdf]

Figure 1: Source Data

D

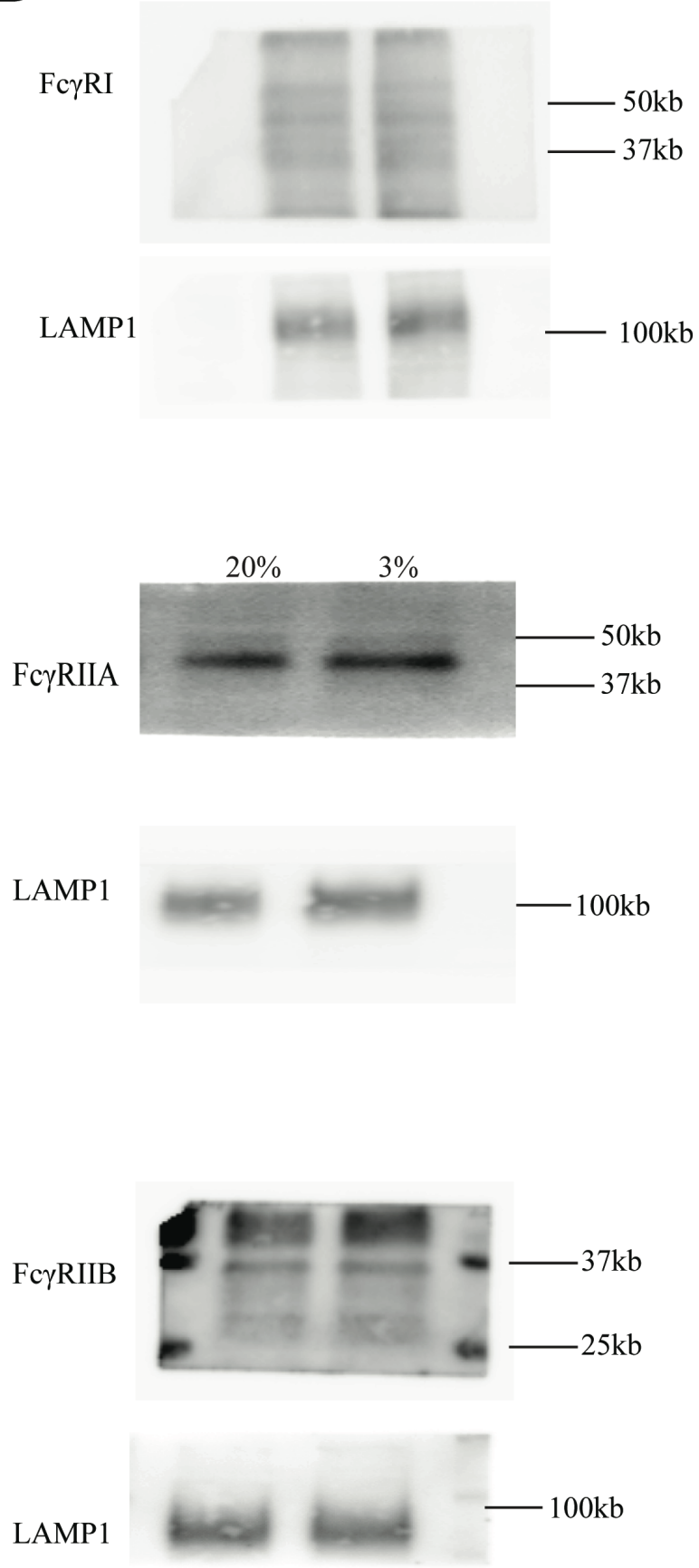

I

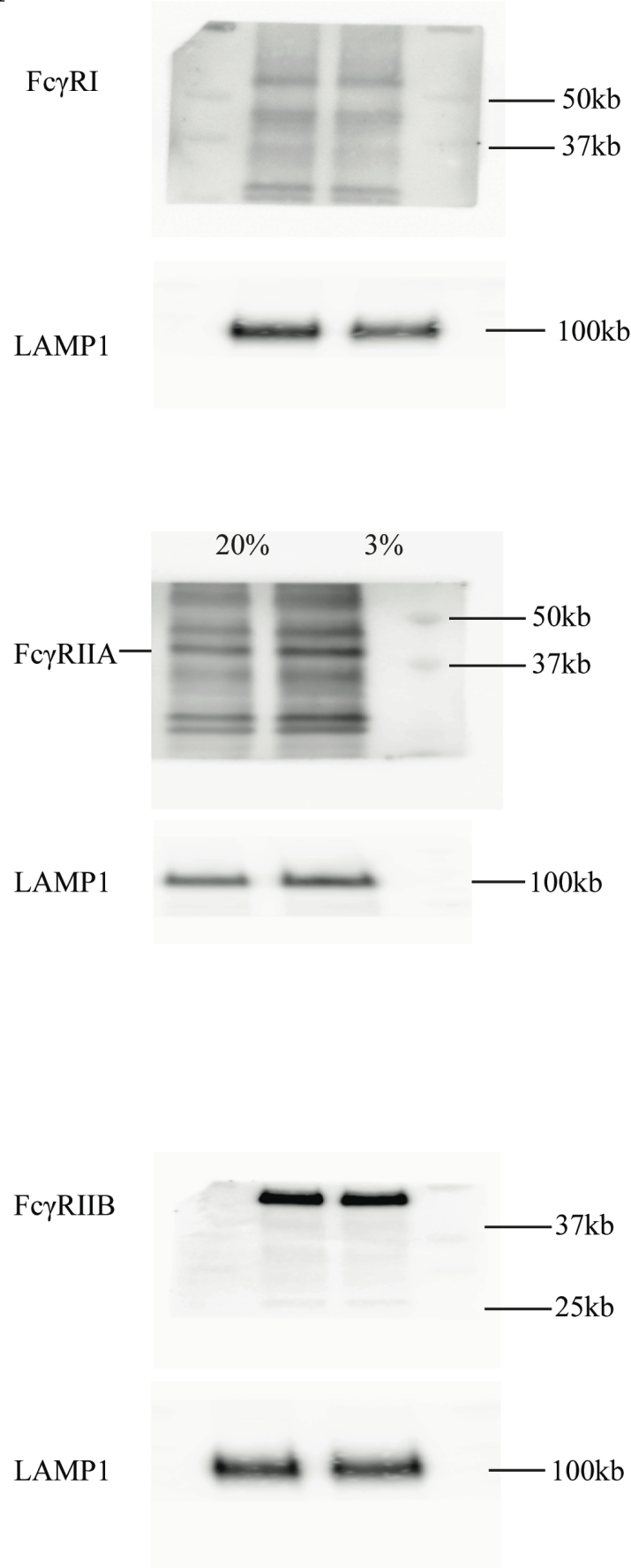

Supplement: Supplementary file 6 — Source Data for Figure 1 [file EMBJ-36-1348-s004.pdf]

Figure 3: Source Data

F

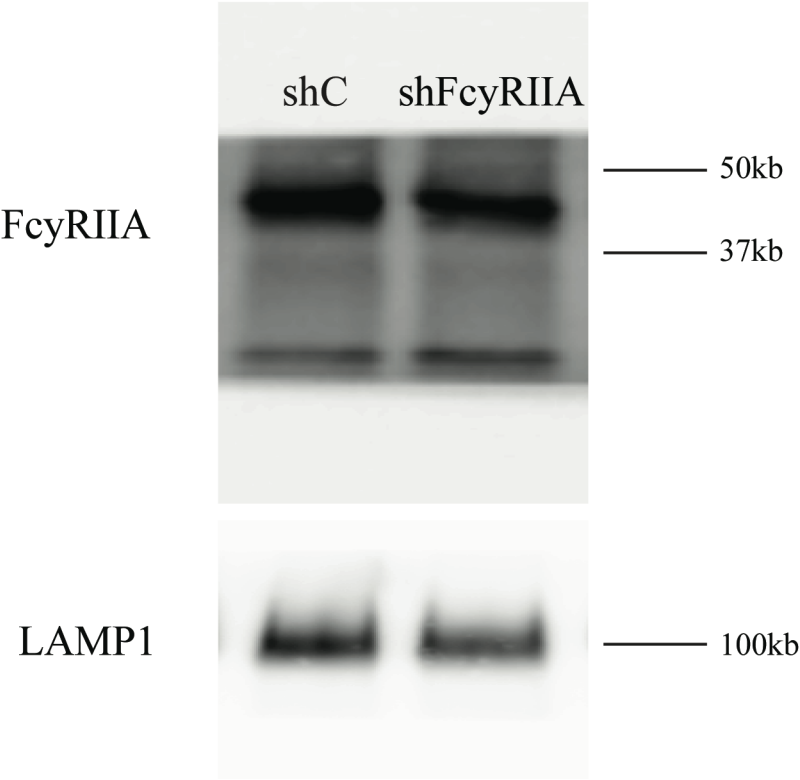

Supplement: Supplementary file 7 — Source Data for Figure 3 [file EMBJ-36-1348-s005.zip › Source_data_for_Figure3/Figure_3_Source_Data.pdf]

Figure 4: Source Data

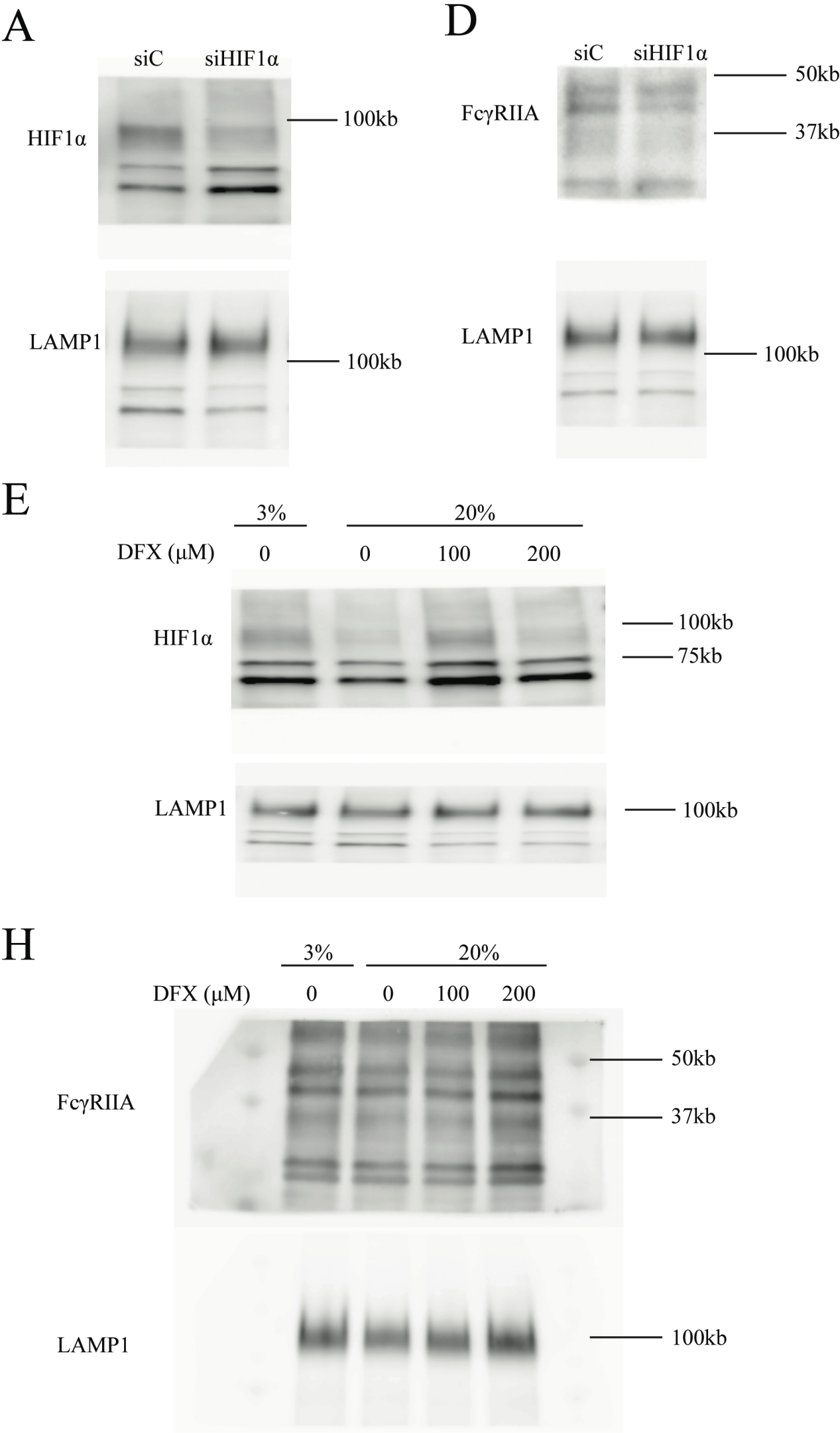

Supplement: Supplementary file 8 — Source Data for Figure 4 [file EMBJ-36-1348-s006.pdf]

Figure 7: Source Data

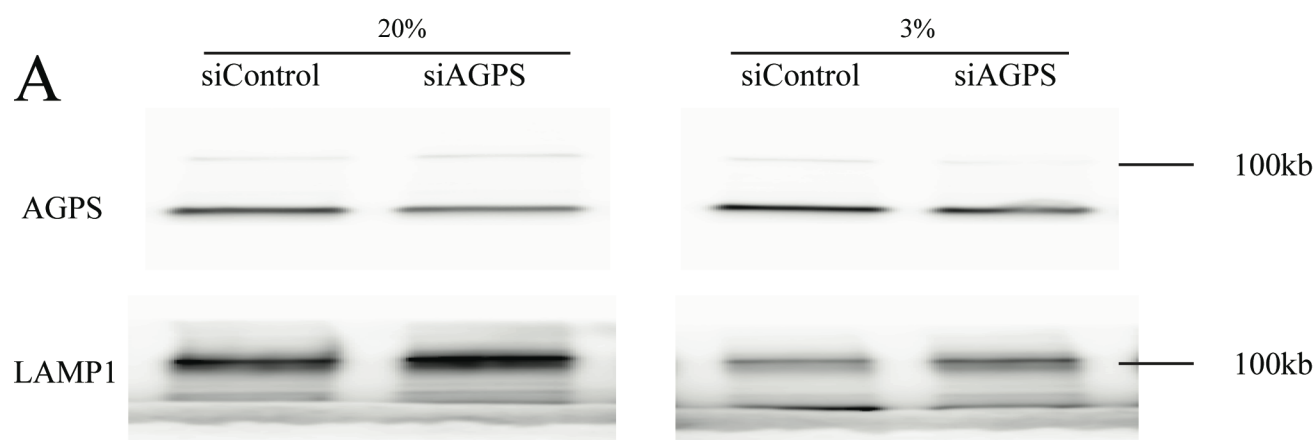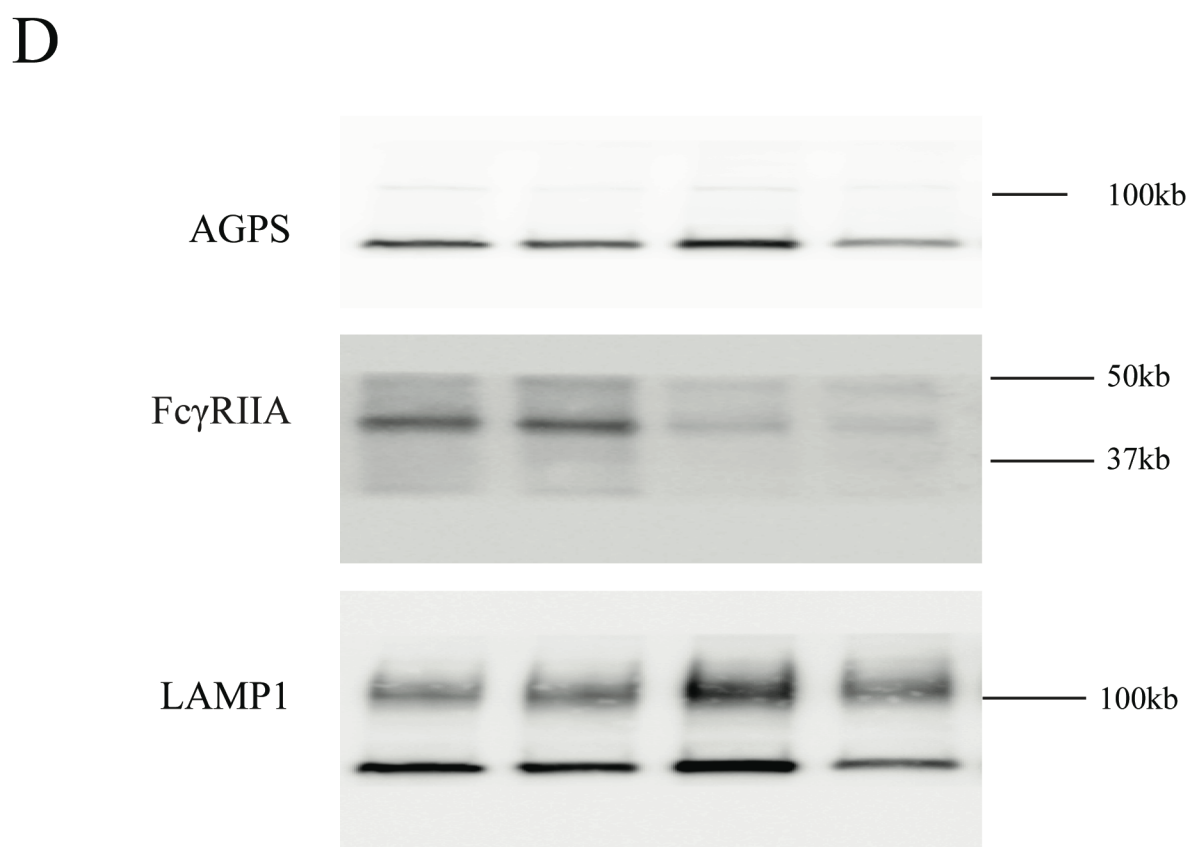

|           |   |   |   |   |
|-----------|---|---|---|---|
| shControl | + | + | - | - |
| siControl | + | - | + | - |
| shFcγRIIA | - | - | + | + |
| siAGPS    | - | + | - | + |

Supplement: Supplementary file 9 — Source Data for Figure 7 [file EMBJ-36-1348-s007.pdf]
