# Supplementary material for: Hypoxia enhances antibody‐dependent dengue virus infection
Source: EMBO J. 2017 Mar 20;36(10):1348–63. doi: 10.15252/embj.201695642 (PMC5430213; doi:10.15252/embj.201695642)
Supplement: Supplementary file 7 — Source Data for Figure 3 [file EMBJ-36-1348-s005.zip › Source_data_for_Figure3/Source_Data_Figure3_legend.docx]

**Movie EV2: Imaris spot analysis of FcγRIIA internalization with DENV immune complexes.**

Hypoxic THP-1 cells were infected with h3H5-opsonized DENV and imaged 1-hour post infection. After staining for surface FcγRIIA (blue), total FcγRIIA (red) and DENV (green) (as per Fig3D), Imaris 8.1.2 spot analysis algorithm was used to visualize the center of each of the blue, red and green spots. Red and green spots that were not colocalized within 100nm with each other or with blue dots were then removed from the analysis. Co-localization of red and green spots were then identified at 6x magnification and with tilting of the image to better visualize internalized DENV with FcγRIIA. The last frame of the video is shown as an image in Fig 3E.
